# Parahippocampal–ventromedial prefrontal cortex functional coupling mediates the association between subjective time perception and delay discounting

**DOI:** 10.1038/s41598-025-95350-x

**Published:** 2025-03-24

**Authors:** Hyun Jung Han, Wi Hoon Jung

**Affiliations:** Department of Psychology, Gachon University, 1342 Seongnam-daero, Sujeong-gu, Seongnam-si, Gyeonggi-do 13120 South Korea

**Keywords:** Delay discounting, Functional connectivity, Parahippocampal cortex, Ventromedial prefrontal cortex, Time perception, Behavioural methods, Imaging, Biomarkers, Medical research, Neurology, Risk factors

## Abstract

Delay discounting (DD) refers to a decrease in the perceived value of an outcome when its delivery is delayed. Time perception (TP), the subjective awareness of the passage of time, is considered a critical factor enabling the modification of heightened DD. However, little is known about the neural mechanisms mediating the relationship between subjective TP and DD. To address this, we instructed participants to perform the TP task while watching scenic videos moving leftward or rightward, followed by the DD task, during brain scanning. We observed that subjective TP became faster while watching rightward scenic movies [the left-to-right (LtoR) condition, which is the participants’ native language reading direction] compared to baseline, and there was a significant correlation between differences in subjective TP and DD between the LtoR condition and baseline. Seed-based connectivity analyses revealed a relationship between behavioral data, including TP and DD, and left parahippocampal seed connectivity under the LtoR condition. Subsequent mediation analysis revealed that the left parahippocampal seed-ventromedial prefrontal cortex functional connectivity mediated the relationship between subjective TP and DD. Our findings suggest a natural scenic effect on subjective TP manipulation and provide insights into the neural mechanisms mediating the relationship between subjective TP and DD.

## Introduction

Modern human lives are full of decisions between choices linked to deciding between immediate and future rewards. For example, even after many of us decide to go on a diet, we still struggle to stop ourselves from consuming extra chocolate cake, which restricts our appetites. Likewise, individuals tend to value smaller-but-immediate rewards over larger-but-delayed rewards, a phenomenon called delay discounting (DD). In a wide range of decision-making research fields, DD has been investigated by quantifying the rates at which a reward is discounted as a function of time until the receipt of that reward^1,2^. Higher discount rates have been associated with impulsivity, as impulsive individuals choose smaller-but-immediate rewards more often than larger-but-delayed ones. Furthermore, a growing body of literature has revealed a relationship between high DD and psychiatric diagnoses, such as drug addiction, attention-deficit/hyperactivity disorder, and schizophrenia^3–6^. However, a previous study has demonstrated that DD can be modified through behavioral training and manipulation^7^.

Time perception (TP) is a comprehensive mechanism that can modify individuals’ DD (i.e., discount rates)^7,8^. The perceived passage of time differs subjectively between individuals, making it a changeable construct. Depending on what activities people engage in, with whom, or where, some perceive time to pass faster than the objective time, whereas others perceive time to pass slower. In addition, subjective TP varies due to perceptual content; people often judge a faster time duration while watching countryside, office, or café scenes than busy city scenes^9^. Moreover, an individual’s mental representations of time are constructed according to their language, in which direction one reads and writes, with prior research showing that those who have rightward direction habits in their languages develop mental timelines that go from left to right, while the reverse is true for those with leftward direction habits^10,11^. Variabilities in TP can alter the way in which individuals weigh the delayed time as a cost, allowing them to eventually evaluate the time delays as more bearable. For instance, if the perception of time delay is not too long, it can be seen as a not-too-high cost, meaning that individuals are more likely to select delayed rewards. Previous studies have observed successful modifications of discount rates through time-perception manipulations, such as presenting delays as specific dates, or making the future easier to imagine^12–14^. Impaired future thinking is related to heightened DD, whereas a prospective time perspective attenuates the devaluation of delayed future rewards^15–19^. Accordingly, behavioral manipulations that shift subjective TP toward the future to make it more concrete and closer can decrease heightened DD.

In line with the aforementioned successful behavioral manipulations of the TP in DD, the neural regions underlying value-based decision-making and internal timing processing have been shown to overlap^20,21^. Previous DD studies have revealed several involved brain networks, including a valuation network consisting of the cortical-basal ganglia circuit including the ventromedial prefrontal cortex (vmPFC) and the ventral striatum (VS), and orbitofrontal cortex; a cognitive network (sometimes called the salience network) including the dorsolateral prefrontal cortex and anterior cingulate cortex; and an episodic memory and prospection network consisting of medial temporal lobe structures including the hippocampus, parahippocampal cortex, and entorhinal cortex^20,22–24^. Moreover, previous research has shown that corticostriatal-thalamic circuits modulated by the dopamine system are essential for time processing on a scale of seconds^25–27^. Additional critical areas in internal timing include the supplementary motor area (SMA), premotor cortex, parietal cortex, and medial agranular cortex^28–31^. Indeed, previous studies have demonstrated the neural integration of signals from prospective time perspective processing associated with the medial temporal lobe and the valuation processing involved in the vmPFC and striatum^12,20,32^.

**Fig. 1:**
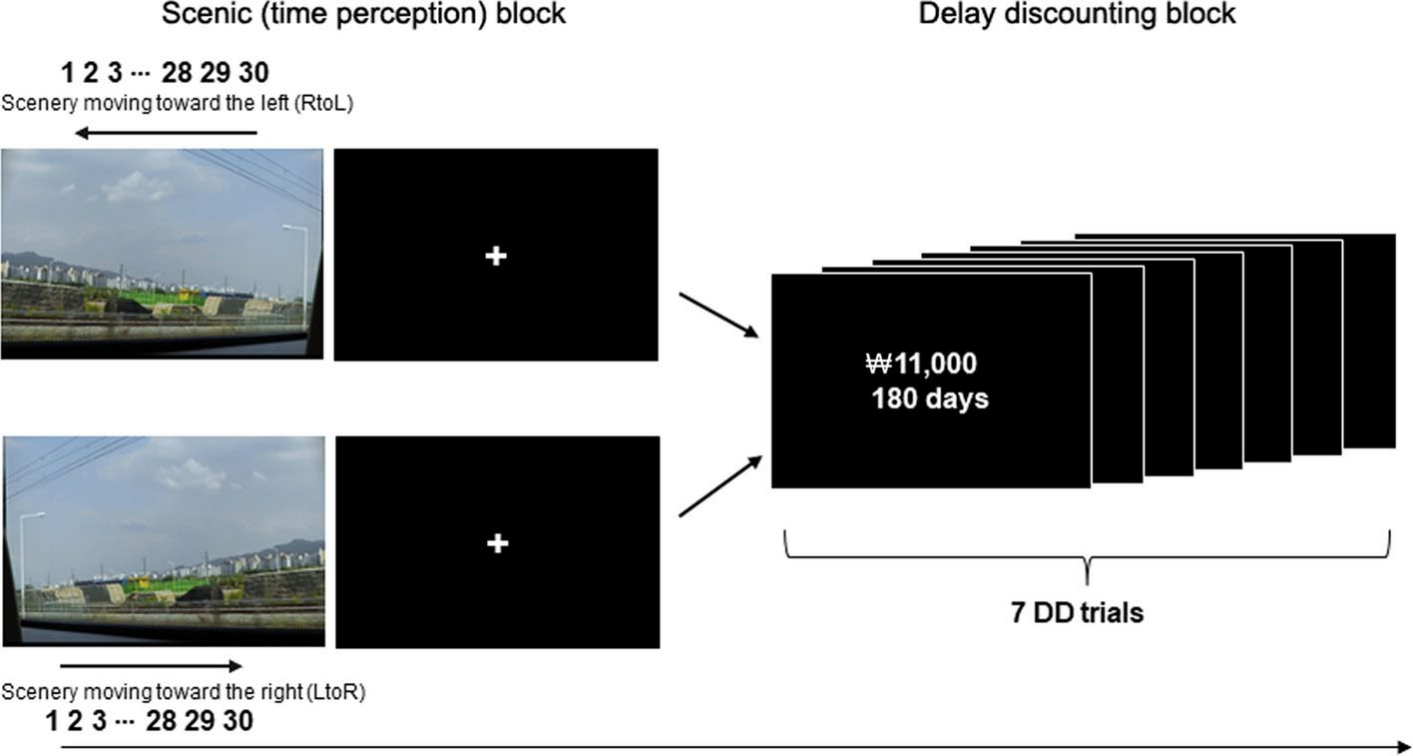
Task layout. The task comprised four runs with two types of blocks: scenic and delay discounting (DD) blocks. During the scenic block, participants performed a time perception (TP) task while passively viewing scenery video clips (40 s). For the TP task, participants had to indicate when they thought a 30-s time span had elapsed by pressing a button. The scenery moved in two directions: left-to-right or right-to-left. After an inter-stimulus interval, seven DD tasks were sequentially presented for 4 s each, yielding a total of 28 s.

Despite the above-mentioned findings that TP influences DD and that brain regions involved in DD and TP overlap, little is known about the neural mechanisms that mediate the relationship between DD and TP. To investigate the neural mediator between these two, we manipulated subjective TP by presenting external stimuli, particularly scenery video clips, and subsequently measured discount rates during brain scanning (Fig. 1). In accordance with previous research on the link between TP and DD, we investigated the following: (1) whether an individual’s sense of time would be modulated by scenic movies, (2) whether the modulated subjective TP would influence subsequent discount rates, and (3) which neural substrates would mediate the effect of the subjective TP on discount rates. To this end, participants were asked to internally count for 30 s while passively viewing one of two types of scenery video clips: those moving toward the left (the RtoL condition) or right (the LtoR condition), after which DD trials were presented sequentially. We predicted that the subjective TP speed would be modulated by external scenic videos, particularly in the LtoR condition, and that the modulated subjective TP would reduce discount rates, while both valuation and prospective neural networks would affect the relationship between TP and DD.

## Results

### Behavioral results

**Fig. 2:**
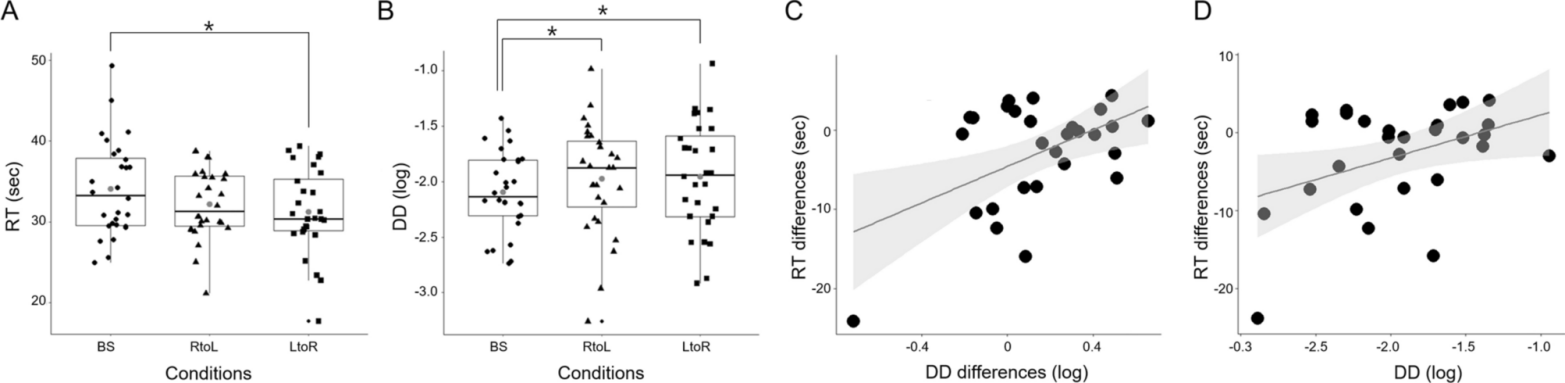
Behavioral results. Data, including response time (RT) from the time perception (TP) task and log-transformed discounting rates (indicated as delay discounting [DD] in the graph above), for baseline (BS) and two different conditions (the left-to-right [LtoR] and the right-to-left [RtoL]) were displayed in different shapes (baseline, circle; the LtoR condition, triangle; the RtoL condition, square). In the graph above, the gray circles represent the mean value across participants. Significant differences are marked with an asterisk (*: *q* < 0.05). (**A**) RTs from the TP task in each condition. The TP task RTs showed a significant difference only in the LtoR scenic block, compared to BS. (**B**) The DD in each condition. The discount rates showed a significant difference in both conditions compared to BS. (**C**) The correlation between differences in the RTs and the following discount rates between each condition and BS. There was a significant correlation between differences in RTs and discount rates only between the LtoR condition and BS (q < 0.05). (**D**) The correlation between differences in RTs (each condition—BS) and discount rates for each condition. The RT differences between the LtoR condition and BS tended to be linked to the discount rates for the LtoR condition itself (uncorrected *p* = 0.032), though this relationship was not significant after correction for multiple comparisons (q = 0.111). This means that in the LtoR conditions, the DD decreased depending on the degree to which TP was changed.

To examine whether an individual’s subjective TP (the TP task RTs) and the subsequent discount rate was modulated by external stimuli, paired sample t-tests were conducted to compare the behavioral data in each condition (LtoR or RtoL) of the scenic block with values collected at baseline. Overall, these comparisons showed that the TP task RTs in the LtoR condition were significantly faster than those in the baseline condition (*t* = − 2.188, *q* = 0.050), but not between the RtoL condition and baseline (*t* = − 1.643, *q* = 0.112) (Fig. 2A). We further observed that the discount rates in both conditions were greater than the baseline (between RtoL and baseline, *t* = 2.269, *q* = 0.050; between LtoR and baseline, *t* = 2.4826, *q* = 0.050) (Fig. 2B), suggesting that watching scenery may modulate subsequent DD.

We subsequently investigated whether this scenery-modulated subjective TP affected subsequent discount rates. To this end, correlation analyses were conducted with either the TP task RTs and discount rates for each condition, or the differences between each of the scenic conditions and the baseline (i.e., the degree of modulated effect). A significant correlation was finally observed between the differences in RTs and discount rates between the LtoR condition and baseline (*r* = 0.501, *q* = 0.047; Fig. 2C), but not between the RtoL condition and baseline (*r* = 0.004, *q* = 0.982). For each condition and at baseline, there were no significant correlations between TP task RTs and discount rates (for the RtoL condition, *r* = 0.164, *q* = 0.667; for the LtoR condition, *r* = 0.0.204, *q* = 0.667; and at baseline, *r* = − 0.033, *q* = 0.982).

To further investigate how the RT differences between each condition and at baseline were related to subsequent discount rates, additional correlation analyses were performed between the RT differences (RTs for each condition and RTs at baseline) and discount rates in each condition. The RT differences (between the LtoR condition—baseline) tended to be linked to the discount rates in the LtoR condition itself (*r* = 0.407, *q* = 0.095; Fig. 2D), though this relationship was not significant after correction for multiple comparisons. In the RtoL condition, there was no correlation between RT differences and discount rates (*r* = 0.140, *q* = 0.667).

To sum up, our findings suggested that the subjective timer may be able to be faster by externally presented scenery stimuli, particularly in the LtoR direction, and that this modulated subjective representation of TP may lead to a decrease in the subsequent discount rates.

### Whole brain results

**Fig. 3:**
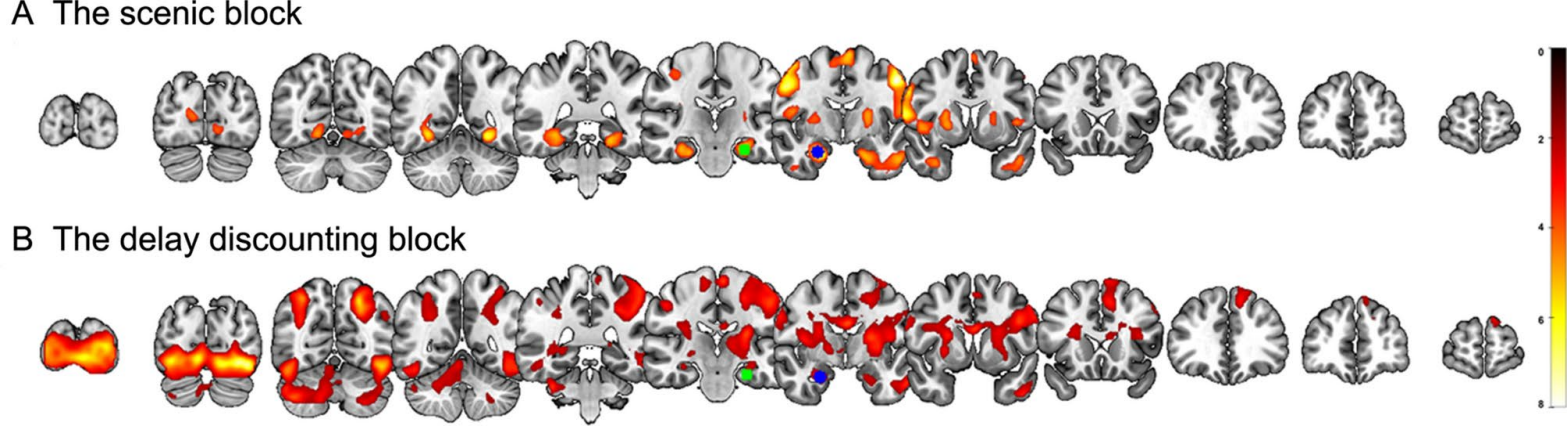
The whole-brain voxel-level activations in each block. (**A**) During the scenic block, there were widespread activations in regions involved in visual scenic processing, including the bilateral parahippocampal cortex, hippocampus, fusiform and lingual gyrus, as well as regions involved in timing, including the precentral, putamen, and supplementary motor area (SMA) across all conditions. (**B**) During the delay discounting block, broad activations were observed in the left fusiform, inferior temporal cortex, right pre- and post-central, left SMA, a large cluster covering the left medial and superior frontal, and bilateral superior parietal regions extending to the inferior parietal, insula, putamen, hippocampus, and parahippocampus across all conditions. For illustration purposes, the left (green) and right (blue) parahippocampal seed regions used for seed-based connectivity analyses are overlaid.

We identified whole-brain voxel-level activation in each of scenic and DD blocks [voxel-level height threshold of *p* < 0.001, uncorrected, corrected for multiple comparisons using a cluster extent threshold of *p* < 0.05, family-wise error rate (FWE)-corrected; Table 1 and Fig. 3]. During the scenic block across all conditions, widespread activation was observed in regions involved in visual scenic processing, including the parahippocampal cortex, hippocampus, fusiform, and lingual gyri, as well as in regions involved in timing, including the precentral gyrus, putamen, and SMA (Fig. 3A). Between-conditions comparisons revealed that the left inferior parietal cortex (x, y, z = − 52, − 28, 42, *Z* = 4.38) was higher in the LtoR condition than in the RtoL condition. During the DD task block across all conditions, broad activation was observed in a large cluster covering the left medial and superior frontal cortices, left fusiform, inferior temporal cortex, right precentral and postcentral cortices, left SMA, and bilateral superior parietal regions, extending to the inferior parietal cortex, insula, putamen, hippocampus, and parahippocampal cortex (Fig. 3B). In between-condition analysis, the left calcarine cortex (x, y, z = − 4, − 96, 12; *Z* = 4.91) was more activated in the RtoL condition, compared to the LtoR condition.

**Table 1:** Regions showing a significant activation in each block.

Block	Brain regions	MNI coordinates	*Z*
During the scenic block			
All conditions	L precentral	− 54, − 2, 46	5.70
	R precentral	54, − 2, 40	5.58
	L parahippocampal & hippocampus	− 26, − 18, − 22	4.97
	R parahippocampal & hippocampus	24, − 6, − 24	5.15
	L fusiform	− 26, − 52, − 10	5.06
	L putamen	− 22, 0, 10	4.92
	R putamen	24, 2, 6	4.54
	L SMA	− 4, 0, 66	4.75
	R SMA	10, − 2, 62	4.85
	R lingual	14, − 66, − 6	4.14
LtoR > RtoL	L inferior parietal	− 52, − 28, 42	4.38
During the DD block			
All conditions	L fusiform	− 40, − 78, − 14	7.55
	L superior parietal	− 24, − 66, 48	6.56
	R superior parietal	32, − 64, 52	5.97
	R postcentral	64, 0, 30	5.82
	R precentral	14, − 20, 64	4.08
	L SMA	− 8, 16, 52	4.83
	L medial superior frontal	− 12, 28, 48	4.99
	L inferior temporal	− 66, − 16, − 20	4.83
RtoL > LtoR	L calcarine	− 4, − 96, 12	4.91

This table lists the peaks and local maxima.

L: left; R: right; SMA: supplementary motor area; LtoR: left-to-right; RtoL: right-to-left; DD: delay discounting; MNI: Montreal Neurological Institute.

Whole-brain multiple regression analyses were performed to confirm the relationship between behavioral data and neural activation in each block (height threshold of *p* < 0.001, uncorrected, combined with extent threshold of *p* < 0.05, FWE-corrected). As a result, for the scenic block across all conditions, the activation in the inferior parietal cortex, an area overlapping with the cluster identified in the LtoR versus RtoL contrast during the scenic block, was found to be negatively correlated with the RTs from the TP task (left inferior parietal, x, y, z = − 46, − 44, 52, *Z* = 5.90; right inferior parietal, x, y, z = 54, − 32, 56, *Z* = 4.06); that is, participants with faster internal representation during the TP task showed increased activity in the bilateral inferior parietal region. During the DD task block across all conditions, activation in the left striatum, including the caudate and putamen, was positively correlated with discount rates, indicating that higher neural activity in these regions is related to being more impulsive (left caudate, x, y, z = − 18, − 6, 20, *Z* = 4.15).

### Seed-based connectivity results

The SBC analysis was performed specifically using the left and right parahippocampal cortices and inferior parietal area, identified from the abovementioned GLM analyses, as seed regions of interest^33–35^. This is because these regions are activated in the subjective TP while watching scenery videos, are involved in selective responses to visual scenic processing^36^, and are part of a prospective network^20,23^.

**Fig. 4:**
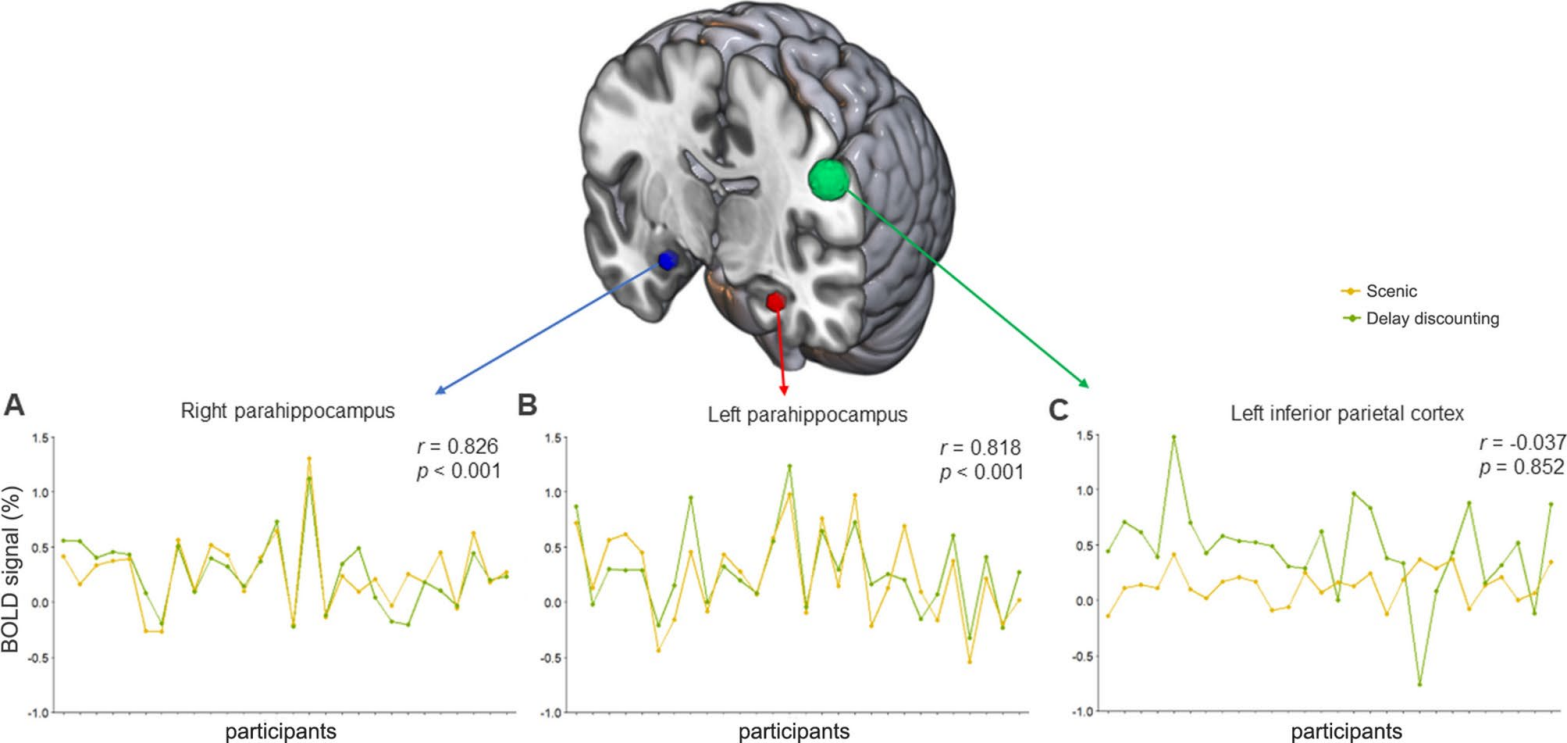
Correlations between mean blood-oxygen-level-dependent signal time series extracted from each seed region in each block across participants. (**A**, **B**) A high correlation can be observed between the mean time series extracted from for the parahippocampal seed regions for the scenic block and the delay discounting block, suggesting that the regions play an important role in both blocks. (**C**) However, the time series of the left inferior parietal region was not correlated between each block.

To determine brain regions in which responses in the scenic blocks influence responses in subsequent DD blocks, we investigated whether there was a relationship between the patterns of neural activity in the scenic and DD blocks in each seed region (the left and right parahippocampal cortices and the left inferior parietal cortex) across participants [q < 0.05, false discovery rate (FDR)-corrected; Fig. 4]. Consequently, we found a high correlation between the time series of the parahippocampal areas extracted from the scenic block and the subsequent DD task block across participants (left parahippocampus, *r* = 0.818, *q* = 0.001; right parahippocampus, *r* = 0.826, *q* = 0.001) (Fig. 4A, B). However, those in the left inferior parietal region did not correlate between the two blocks (*r* = − 0.037, *q* = 0.852; Fig. 4C).

Based on the above-mentioned results, whole-brain multiple regression analyses were performed between behavioral data and seed-based connectivity (SBC) maps generated by the left (right) parahippocampal seeds (height threshold of *p* < 0.001, uncorrected, combined with extent threshold of *p* < 0.05, FWE-corrected). These analyses revealed that the functional connectivity (FC) between the left parahippocampal seed and left vmPFC (x, y, z = − 10, 40, − 6, *Z* = 4.74) were significantly negatively correlated with the RTs from the TP task in the LtoR condition. Furthermore, the FC between the left parahippocampal seed and bilateral inferior parietal cortex (left, x, y, z = − 64, − 32, 34, *Z* = 4.21; right, x, y, z = 66, − 40, 40, *Z* = 4.37) exhibited a significant positive correlation with discount rates in the LtoR condition.

### Mediation analysis results

**Fig. 5:**
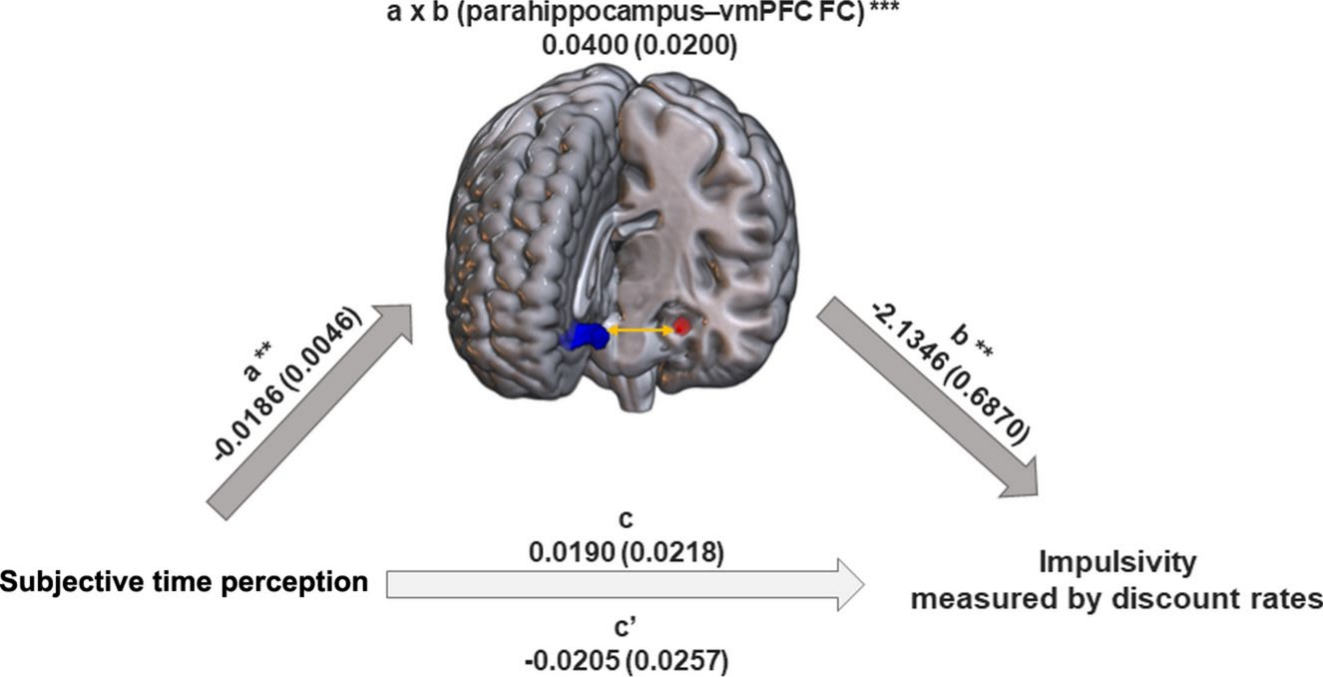
Mediation analysis results. The functional interaction between the parahippocampal and ventromedial prefrontal cortex (vmPFC) mediated the relationship between response times (RTs) from the time perception task and discount rates in the left-to-right condition. In particular, RTs were negatively correlated with the parahippocampal–vmPFC functional connectivity (FC) (*path a*). This FC was also negatively correlated with discount rates (*path b*). Finally, this parahippocampal-vmPFC FC showed a positive mediation effect (positive path a*b).

As the neural system that modulates the association between subjective TP and discount rates in the LtoR condition, the FC between the left parahippocampal seed and left vmPFC fully mediated the relationship between RTs from the TP task and discount rates (Fig. 5). In particular, TP task RTs were negatively correlated with parahippocampal–vmPFC FC (*path a*). This FC was also negatively correlated with discount rates (*path b*). Finally, parahippocampal–vmPFC FC showed a positive mediating effect (positive path a*b). This mediation result can be interpreted as suggesting that individuals whose internal timer ‘sped up’ while watching the rightward scenery movies (the LtoR direction) showed strengthened functional coupling between the parahippocampus and vmPFC, and that this stronger FC brings lower impulsivity.

## Discussion

In this study, we investigated the natural scenic effect on subjective TP manipulation and its beneficial impact on discount rates. We observed that scenic movies, particularly those moving toward the right (the LtoR condition), modulated individuals to perceive a 30-s interval shorter than the objective time. In the LtoR condition, compared to the baseline, the degree of differences in the TP task RTs was linked to the degree of the differences in the following discount rates, indicating that the more scenery was modulated, the less impulsive the participants were. Next, we investigated the neural mechanisms underlying subjective TP and discount rates. Overall, the SBC results highlighted the enhanced functional coupling between the parahippocampus and vmPFC in individuals whose internal timer was modulated faster by rightward moving scenic videos. Mediation analyses further showed that parahippocampal-vmPFC FC fully mediated the relationship between subjective TP and discount rates. These findings provide novel insights into the functional interaction between the parahippocampal cortex and the vmPFC, accounting for the impact of subjective TP on impulsivity.

At the behavioral level, consistent with previous studies^9,37,38^, the current study showed that viewing landscapes can influence subjective TP. Several recent studies have argued that our perception of time can be distorted depending on what we look at^9,37,38^. In the behavioral data of this study, although only the LtoR condition, compared to the baseline, was statistically significant, subjective TP showed faster values (smaller RT values) on average in both directions than the baseline. It is speculated that watching the scenery may be the primary factor that greatly influences TP, and the direction in which the scenery moves may have a secondary influence. Statistical analysis revealed that subjective TP was modulated to be faster by the right-moving scenic videos compared to the baseline. However, this modulated effect was not observed when watching videos of leftward-moving scenery compared to the baseline. This finding could be explained by the function of reading and writing directions^39^. Several previous studies have shown that directional reading habits influence cognitive processing^40–42^. Native Korean participants, who utilize a left-to-right orthographic system, probably prefer the rightward movement condition. The habitual direction of perceptual scanning can facilitate cognitive processing. This result may therefore be interpreted as follows: exposure to scenery moving in an individual’s habitual reading direction facilitates one’s abstract conceptual representation of the movement as a sense of time, thus modulating the perceive a 30-s time duration passing faster than a stopwatch. Moreover, the degree of differences in RTs from the TP task between the LtoR condition and baseline was found to be correlated with the degree of differences in the following DD, indicating that the greater the extent of RT differences between the LtoR condition and baseline, the lower the discount rates observed. Although there is currently no consensus regarding the relationship between subjective TP and impulsivity, people who perceive certain periods of time as being longer than they actually are may be more likely to perceive delayed time intervals in the DD task as subjectively lasting too long^8,36,43,44^. Therefore, our behavioral results suggest that neural substrates may connect behavioral subjective TP and DD.

At the neural level, our whole-brain imaging results during the scenic block were consistent with previous findings^25,27,28^. Medial temporal regional engagement, including the parahippocampal and hippocampal regions, and the occipital visual cortex, precisely mirrored the external visual stimulation of scenic movies. As the parahippocampal area responds more strongly to visual scenes, activation of this region contributes to the responses of watching scenic videos^45^. Furthermore, the activation was extended to the hippocampus, implying that our instruction of subjects to imagine sitting in a train chair and watching landscapes would be likely to trigger their episodic memory or prospective processing^20,46^. In line with prior studies on temporal processing, the involvement of the frontostriatal network, consisting of the SMA, insula, and striatum, reflects neural engagement in performing time-production tasks^26,27^. Moreover, in the current study, activation in the left inferior parietal cortex was found to only be increased in the LtoR condition not in the RtoL condition, while activation in the region was also negatively correlated with the RTs from the TP task. The inferior parietal lobule is known to have orthographic/phonological representation, as well as number and mathematics processing^47–53^. As such, we speculated that engagement in the left inferior parietal lobule reflects a preference for rightward scenery video clips in our sample with rightward reading directional habits, and that this preferred representation might unconsciously facilitate a subjective sense of time or numbering process.

Several brain regions were found to be involved in the DD task blocks, including the medial superior frontal lobe; striatum covering the putamen, pallidum, and caudate; medial temporal region surrounding the parahippocampus and hippocampus; superior temporal areas extending to the insula and cuneus; superior parietal lobe; occipital visual cortex; and a large cluster containing the SMA and pre/postcentral gyrus. The observed regions were covered with well-known DD-related networks, consistent with previous studies^2,20,21,23,54^. Interestingly, in the current study, a cluster containing the medial temporal lobe consisting of the parahippocampal cortex and hippocampus was observed, with highly correlated fluctuations in the time series between the scenic and DD blocks (see Fig. 4). This finding suggests that parahippocampal activation in response to previously-presented scenery video clips may trigger episodic memory, and this activation may subsequently influence DD. Multiple regression analysis showed that the striatum, including the caudate and pallidum regions, was positively correlated with the discount rate under all conditions. Several studies have also identified the engagement of the striatum, including the caudate, putamen, and VS, to respond to temporally discounted values, and an increase in the activation or volume of this region was correlated with more significant discount rates^55–57^.

Our findings from the SBC and mediation analyses highlight the neural involvement of individual variations in timing and discount rates. For example, we found that the parahippocampal-vmPFC FC fully mediated the behavioral relationship between scenery-modulated subjective TP and discount rates. This full mediation indicates that subjective TP influences discount rates indirectly through the neural pathway between the parahippocampal cortex and vmPFC, rather than having a direct effect. The finding supports the idea that decision-making related to temporal discounting is not solely based on valuation (vmPFC) but also involves contextual and memory-based processing (parahippocampal cortex). Although there were no instructions to induce autobiographical memory retrieval during the entire study, we may infer that the participants’ imaginations, as if watching the natural landscape out of the train window, contributed to triggering their autobiographical memory. Consequently, we considered that episodic and prospective effects would have integrated the neural networks underlying episodic/prospective and valuation processes^4,12,20^. The imagery and prospective system consisting of medial temporal lobe structures, the parahippocampal cortex and hippocampus, is believed to be responsible for “future thinking” in the DD task^20,22,58^. In addition, the vmPFC depends on a future-time perspective by valuing future outcomes^15^. Therefore, the connection between these two regions suggests that memory or contextual information (parahippocampal cortex) may influence decision-making related to reward valuation (vmPFC). In other words, this connection may be involved in integrating contextual memory with valuation processes to guide decisions about rewards and delays. In agreement with the functions of these two brain structures, our mediation analysis revealed that individuals who produced a shorter time interval than a stopwatch may have had strengthened functional coupling of the parahippocampus–vmPFC, suggesting that enhanced functional coupling reduces impulsivity. Therefore, it is suggested that individuals with stronger FC between the parahippocampal cortex and vmPFC may be able to integrate temporal information more effectively. The observed parahippocampal-vmPFC FC may reflect the neural mechanisms by which TP shapes intertemporal decision-making, allowing individuals to make future-oriented choices based on subjective time estimates.

This study has several limitations. First, all the participants were young, healthy college students. This restricted sample population may have limited the generalizability of our findings, as differences in age, sex, and educational factors may result in differences in TP and discount rates. Future research should extend sample selection. Second, in the behavioral results with the TP task RTs and discount rates, significant differences in RTs were observed in the LtoR condition compared to the baseline, but there were no significant differences between the two conditions (LtoR and RtoL; *p* > 0.05). Moreover, the discount rates showed a significant difference in both conditions compared with the baseline, raising the question of whether the observed behavioral results are due to differences in circumstances between the MRI scans and baseline rather than the presented scenic videos. For example, the noise generated during an MRI scan (which has a certain rhythm) may affect the TP and/or DD. However, we cannot completely eliminate the possibility that these factors affected the results. Nevertheless, based on our correlation results, a significant correlation between differences in RTs and discount rates was only observed between the LtoR condition and baseline, as expected. Additionally, previous studies have demonstrated that specific movements in space (e.g., observing people moving virtually backward rather than forward through space) may affect temporal movements^37^. Future studies should measure baseline values in the same MRI environment (i.e., specific blocks to perform the TP and DD tasks without watching scenic video clips, rather than simply the rest period, during brain scanning) to reduce the differences between the baseline and task states. Finally, as mentioned above, because the baseline behavioral data in the current study were measured outside the MRI, we cannot directly compare the behavioral and brain responses for the task performed while watching the scenic landscape with those of the task performed without watching the scenery as a baseline condition. In other words, in the current study, it was not possible to investigate changes in neural mechanisms in the scene condition and at baseline. However, our mediation results in the LtoR condition showed that the parahippocampal–vmPFC FC fully mediated the relationship between scenario-modulated subjective TP and subsequent DD. Based on our results showing significant differences in behavioral data between the LtoR condition and baseline, we suggest the RT value for the LtoR condition itself may be the value that was influenced and changed by the scenic stimuli, whereas the difference between the LtoR condition and baseline represents the amount of change modulated. The results of the current study should be confirmed in future studies with a larger sample size directly comparing neural responses from scene conditions and the baseline, which are measured in the same MRI scanning environment.

In conclusion, our results show that natural scenic stimuli moving in the same direction as an individual’s habitual reading direction may cause subjective internal timers to become faster, suggesting that variabilities in subjective TP may serve as a beneficial tool for manipulation to reduce impulsivity. Additionally, our findings suggest that the modulated subjective TP works in harmony with neural networks of the parahippocampal cortex, part of the prospective network, and the vmPFC, part of the valuation network, to revalue the rewards and lead to making less impulsive decisions. As such, our findings on the neural mechanism mediating subjective TP on impulsivity observed in the current study provide important implications for mental health by developing interventions to manipulate subjective TP and may also help us make better decisions by maximizing future payoffs.

## Methods

### Participants

Thirty-two healthy young adults were recruited for this study. Among them, four were excluded because of a lack of data points due to failure to complete the task (n = 1), or excessive head motion during the task (n = 3). Overall, 28 participants (15 males, 13 females; age [mean ± SD], 22.39 ± 3.06 years; the duration of education, 15.11 ± 1.34 years) were analyzed. We checked the Mahalanobis distance for outlier detection in terms of behavioral data in this dataset, but there were no outliers. All participants had normal to corrected-to-normal vision and no significant medical illnesses. Written informed consent was obtained from all participants in accordance with the Declaration of Helsinki. All experimental protocols were approved by the Institutional Review Board of Daegu University prior to data collection.

### Experimental design

The task design is illustrated in Fig. 1. The task comprised four runs with two types of blocks: a scenic block (the TP task block) and a DD block. The presentation of the scenic block was followed by the DD block. That is, these two types of blocks were presented alternately in each run. During the scenic block, participants performed the TP task while passively viewing scenery video clips. They were further told to imagine watching the scenery outside the window of a moving train. At the same time, they performed the TP task, during which they had to press a button when they thought a 30-s time window had elapsed. The scenery in the clips moved in two directions: left-to-right (LtoR condition, i.e., participants’ native language reading direction) or right-to-left (RtoL condition). Each direction was presented in a run to obtain a sufficiently robust directional effect on the TP task. After one scenery video clip, seven DD trials were presented sequentially. During the DD task, participants were asked to choose between a fixed immediate reward of ₩10,000 (roughly $8 ~ $9 USD) and a larger delayed reward that varied randomly from trial to trial by pressing a button. The value of the larger reward varied from ₩11,000 to ₩60,000, while the delay varied from 2 to 180 days. There were 49 unique choices in each run for 196 trials over four runs. Thus, 14 scenic and 98 DD trials in each direction (LtoR or RtoL condition) were provided over two runs. The order in which each run was presented (i.e., the order of LtoR and RtoL conditions) was counterbalanced. Before scanning, the participants were briefed on the entire task procedure and completed a practice run.

Before scanning, all participants performed the TP task twice, without scenery videos, after which the DD tasks were performed. The acquired response times (RTs) from the TP task were averaged for each participant and used as the baseline. Presentation software (v23.0) was used to present the stimuli and collect behavioral data.

### Behavioral analysis

Consistent with previous findings, the discount curves for all participants fit a hyperbolic model^1,2^. To estimate an individual’s discount rate, we determined the best-fitting discount parameter *k* based on the following formula:

$$SV = \frac{A}{{\left( {1 + kD} \right)}}$$

where *SV* is the subjective value of the delayed reward, *A* is the total amount of the delayed reward, *D* is the delay, and *k* is the discount rate parameter of the individual. Higher *k* values (discount rates) correspond to greater impatience. The discount rates were log-transformed to normalize the distribution before statistical analyses.

Paired t-tests were further performed to compare RTs from the TP task (or discount rates) between each task condition (the LtoR or RtoL condition) and the baseline (Fig. 2A, B). To quantify the degree of the scenery-modulated effect on the TP and DD, we calculated the differences in RTs between each task condition and baseline (for example, RTs during the LtoR [or the RtoL] condition—RTs at baseline). That is, the RT differences were construed as the degree of discrepancies (i.e., the degree of modulated effect) between scenery-modulated subjective time (i.e., RTs in each condition) and the participant’s original subjective sense of time (i.e., RTs at baseline). To determine the relationship between scenery-modulated subjective TP and DD, we further examined whether RT differences were correlated with differences in discount rates or discount rates in each condition using the Pearson correlation method (Fig. 2C, D). The significance of these t-tests and correlation analyses was corrected for multiple comparisons using FDR (q < 0.05).

### Image data acquisition and preprocessing

All imaging data were acquired using a 3 T scanner (Siemens Magnetom Trio; Erlangen, Germany). The functional magnetic resonance imaging (fMRI) images were obtained using a T2*-weighted gradient echo-planar imaging (EPI) sequence (echo time [TE] = 20 ms, repetition time [TR] = 2 s, flip angle [FA] = 90°, voxel size = 3 × 3 × 3 mm^3^, and interleaved axial slices = 42). High-resolution T1-weighted magnetization-prepared rapid gradient echo anatomical images were acquired (TE = 2.52 ms, TR = 1900 ms, FA = 9°, voxel size = 1 × 1 × 1 mm^3^, 192 sagittal slices).

Image preprocessing was performed using SPM12 software (http://www.fil.ion.ucl.ac.uk/spm) that is run in MATLAB (R2022a). All data were corrected for both slice timing acquisition and head motion, after which they were spatially normalized to the standard MNI EPI template. Normalized volumes were resampled into 2 × 2 × 2 mm^3^ isotropic voxels, and spatially smoothed with an 8 mm full-width half-maximum Gaussian kernel.

### Whole-brain analyses

Whole-brain image analysis was performed using SPM12 software. For each participant, a first-level general linear model (GLM) was used to create contrasts for each condition of interest (scenic and DD blocks in each direction, respectively) against implicit rest periods (baseline) (i.e., condition > rest). All regressors, along with the six head-motion parameters, were included after convolution with a canonical hemodynamic response function (HRF) and its temporal and dispersion derivatives. To exclude confounding effects (i.e., the motor effect caused by pressing a button), the duration of the scenic block was defined as the time between the onset of the presented video and 2 s prior to the button press. The duration of the DD block was defined as the time between the onset of the first and sixth trials.

One-sample t-tests were performed for each of the scenic and DD blocks based on individual contrast maps (Fig. 3). For condition-specific neural activity, paired t-tests were conducted to compare two paired conditions (LtoR versus RtoL) for each scenic and DD block. Multiple regression analyses were further conducted to determine the areas associated with behavioral data (RTs from the TP task and discount rates from the DD task) for each scenic and DD block. To correct for multiple comparisons, we applied FWE-corrected *p* < 0.05 for the cluster-level extent-threshold along with *p* < 0.001, uncorrected, for the voxel-level height threshold.

### SBC analysis

The SBC analysis was performed using the CONN toolbox (v21a; https://www.nitrc.org/projects/conn/) pipeline^59^. SBC maps were used to characterize condition-specific FC strength and were computed using a weighted least-squares linear model with user-defined temporal weights for each individual experimental condition (i.e., condition-specific boxcar timeseries convolved with canonical HRF). To correct for the confounding effects of motion and other artifactual noise, a denoising step, involving a component-based correction method, was applied by extracting the principal components related (as noise components) to the segmented white matter and cerebrospinal fluid regions^60^. Noise components were further used as confounder regressors in the first-level GLM. To control for simple condition-related activation effects, we also included task conditions as confounding regressions for SBC. The SBC map was created by calculating condition-specific Pearson correlation coefficients (i.e., FC strength) between the time series of a seed region and those of voxels outside the seed for each block. The correlation coefficients on the SBC maps were converted to z-scores using Fisher’s transformations for second-level analyses.

We were particularly interested in the role of the parahippocampal cortex in modulating subjective TP while watching scenery videos, as this region is known to exhibit a selective response to visual scenic processing^36^, and is part of a prospective network^20,23^. Therefore, we performed SBC analysis on the left and right parahippocampal cortices and inferior parietal areas identified from the abovementioned GLM analyses (see Results; Table 1) as seed regions of interest^33–35^. The size of these seed regions was determined according to the size of the cluster observed in the above-mentioned GLM analyses (see Results; Table 1); that is, we created 4 mm radius spherical parahippocampal seeds centered on the coordinates derived from the contrast of the scenic block (left x, y, z = − 26, − 18, − 22; right x, y, z = 24, − 6, − 24; Table 1) and a 6 mm spherical left inferior parietal cortex seed area centered on the coordinates derived from the contrast of the scenic block (left x, y, z = − 52, − 28, 42; Table 1).

We then investigated whether there was a relationship between the activation patterns (i.e., blood-oxygen-level-dependent signal) extracted from the scenic and DD blocks across conditions in each seed region across participants, to determine whether neural activation in response to the scenic block influenced neural activation during the subsequent DD task block (q < 0.05, FDR-corrected; Fig. 4). We subsequently performed whole-brain voxel-wise multiple regression analyses between behavioral data (RTs from the TP task and discount rates) and SBC maps for each of the left and right parahippocampal seeds in the LtoR condition to explore the neural mechanisms underlying the behavioral effects modulated by external scenic stimuli. Significant regions were reported if they survived the voxel height threshold of *p* < 0.001, uncorrected, and the cluster-level threshold of FWE-corrected *p* < 0.05.

### Mediation analysis

The above SBC results, calculated to show the relationship between behavioral data and left parahippocampal FCs in the LtoR condition (see Results), provided possible insights into the neural mechanisms bridging subjective TP and discount rates. We therefore proceeded with mediation analysis to discover the underlying neural substrate. In other words, to investigate the neural mechanisms underlying the relationship between subjective TP and discount rates in the LtoR condition, we tested whether the effects of TP task RTs (X; *path a*) on discount rates (Y; *path b*) could be indirectly explained by the left parahippocampal seed-left vmPFC FC strength (M; path a*b) as a mediator (Fig. 5). We used the M3 Mediation Toolbox (https://github.com/canlab/MediationToolbox). Bootstrapping with 10,000 resamples was used for the statistical inference of each path (*p* < 0.05).

## Data Availability

The datasets generated and analyzed for the present study are available from the corresponding author on reasonable request.
